# A Sensor to Analyze Fish Freshness: A Virtual Sensor Array Based on an Electrochemical Chemotransistor

**DOI:** 10.3390/s26134306

**Published:** 2026-07-07

**Authors:** Yulia Efremenko, Eya Boughanmi, Vladimir M. Mirsky

**Affiliations:** 1 Nanobiotechnology Department, Institute of Biotechnology, Brandenburg University of Technology Cottbus-Senftenberg, 01968 Senftenberg, Germany; iullia.efremenko@b-tu.de (Y.E.); eya.boughanmi@etudiant-fst.utm.tn (E.B.); 2 Analytical Chemistry and Electrochemistry Lab, Faculty of Science, University of Tunis El Manar, Tunis 2092, Tunisia

**Keywords:** fish freshness, electrochemical chemotransistor, conductometric sensor, chemometrics, ionic liquids, affinity control, virtual sensor array, artificial nose

## Abstract

The introduction of a quantitative definition of fish freshness enables the determination of the remaining storage time of raw fish materials. To measure this value, a virtual array of electrochemical chemotransistor-based chemical sensors was developed. The electrolyte used to electrically connect the four measurement electrodes and the reference electrode was optimized. To achieve the high stability, high electrochemical activity of chemosensitive material, and reversible potential of the silver/silver chloride reference electrode, a chloride-containing ionic liquid and polymeric acid mixture was used as the electrolyte. Polyaniline in different redox states was applied as the chemosensitive material with electrically controlled affinity. First, the sensor was evaluated for trimethylammonium detection, and then it was applied to fish samples. Unlike the response observed for trimethylammonium, the sensor’s response to fish samples exhibited complex, non-exponential kinetics and a non-monotonic dependence on the storage duration of fish samples. To characterize these responses, a set of descriptors was introduced. The storage time was estimated by minimizing the Euclidean distance between the descriptors values obtained from fish samples and those determined during calibration. Based on the quantitative definition of freshness, this approach categorizes the current stage of fish products and predicts the remaining storage duration quantitatively.

## 1. Introduction

Fish is an essential part of the human diet. Therefore, the modern fish and seafood market is part of a global industry involving a wide range of fishery products. However, compared to other food products, fishery products have a very short shelf life. For this reason, consumers require more information about the quality of fish and seafood. Over the years, fish processing specialists have attempted to establish basic guidelines to predict the condition of fish and seafood and how long they can be stored. Nevertheless, the main challenge is the lack of objective, clear, and quantifiable criteria with which to accurately evaluate changes in raw fish materials. This issue is further complicated by the fact that core concepts such as “fish quality” and “fish freshness” lack strict scientific and regulatory definitions, leaving them open to subjective interpretation [1,2,3].

Initial fish spoilage begins with enzymatic and chemical processes. Obvious spoilage, on the other hand, is caused by the activity of spoilage microorganisms and is dynamic. After catching the fish, oxygen deficiency occurs in their muscles, transitioning them from aerobic to anaerobic metabolism. This transition results in the acidification of the tissues [4]. At a certain pH level, key glycolytic enzymes are inhibited, which terminates glycolysis. This state is known as rigor mortis. The onset and extent of rigor mortis depend on fish species, environmental factors, and harvesting methods. Further loss of freshness is related to endo-enzymatic reactions (e.g., glycolysis, proteolysis, and lipolysis), an increase in microorganisms, and the accumulation of breakdown products formed in the raw material during storage. Bacterial spoilage depends on the presence of particular types of bacteria, the species of fish, and external ambient conditions [5,6,7].

Due to the obvious importance of environmental factors, an exact definition of storage conditions is required to introduce a quantitative scale of freshness. So far, only integral attributes of freshness, which characterize changes that occur during the post-mortem period, have been applied [8,9]. A quantitative definition of freshness requires the specification of standard storage conditions. The storage temperature is the main environmental factor that determines the rate at which fish products degrade. For the purpose of defining freshness, a temperature of +5 °C was determined. The consortium of the European project FishEUTrust, which comprises 22 European fish science, industry, and trade organizations, approved this definition of the standard storage conditions required for the quantitative freshness characterization. This definition is useful for acquiring calibration data in experimental studies. The result of the freshness analysis indicates that the sample’s state corresponds to storage under standard conditions (+5 °C). It should be noted that this definition allows one to quantitatively estimate not only the current state of fish samples but also the remaining storage time based on comparison with calibration performed under standard conditions: even if the real storage temperature is different, one can say that this particular product has the rest storage time of, i.e., 40% at such conditions. This is of primary importance for developing an optimal retail strategy and setting prices.

Initial attempts to define freshness were based on subjective ratings. The most common method is sensory evaluation. One such method is the “EU scheme,” which is often used in European countries [10] and provides four quality grades (from excellent to condemned). A more objective and accurate method, the quality index method (QIM), has been suggested and established for a series of European fish species [11,12]. The European Research Institutes of Fisheries consider the QIM a scientifically based, objective, organoleptic method for estimating fish freshness [13].

Quantitative analysis of freshness requires instrumental methods. The most common indicators for quantitatively evaluating fish freshness are total volatile basic nitrogen (TVB-N) [14,15] and the K-value, which represents the percentage of inosine and hypoxanthine in the total amount of ATP and its breakdown products [16,17]. Thus, the K-value indicates the extent of ATP degradation: the lower the K-value, the fresher the fish. However, this analytical approach requires high-performance liquid chromatography, which is a time-consuming procedure that requires laboratory conditions [18,19]. For this reason, efforts mainly focus on determining TVB-N. TVB-N consists of amines that accumulate in fish as it loses its initial freshness due to endogenous and bacterial enzyme reactions [20,21]. Trimethylamine (TMA), a TVB-N component, is an important indicator because its presence signals the initiation of microbiological degradation of the fish. [22,23]. For this reason, TVB-N is often considered as a marker of fish freshness. The threshold values for certain fishery product categories and the corresponding analysis methods are regulated by Commission Regulation (EU) No 2074/2005 [24]. Specifically, this regulation establishes strict TVB-N thresholds for fresh fish to be considered fit for human consumption. These thresholds range from 25 to 35 mg of nitrogen per 100 g of fish flesh depending on the species. However, it should be noted that the presence of TVB-N, particularly TMA, indicates that spoilage has already begun, necessitating an approach to determine fish freshness at an earlier stage.

Various chemical sensors and biosensors have been proposed to detect markers of fish spoilage and post-mortem changes. An enzymatic biosensor that detects inosine and hypoxanthine, which result from ATP degradation in fish, was reported in [18]. TVB-N analysis was performed using colorimetric solid sensors based on bromocresol green [14], a colorimetric sensor based on a color change in a copolymer [25], a colorimetric sensor array [14,15], and gas sensors [26,27,28]. These methods were used to monitor levels of this freshness indicator in the headspace of fish. Due to the high volatility of TVB-N, which consists of ammonia, dimethylamine, and trimethylamine, one can analyze fish headspace to determine freshness [29,30]. Polyaniline, a known chemosensitive material for ammonia [31,32], was used for TVB-N detection [33]. Most other materials suggested for ammonia detection could probably be used for this purpose as well, and many have already been tested for this application [34]. More selective approaches are based on the application of electronic noses. Different receptors applied in these systems include tetraphenylporphyrins with different metals [35], commercial or designed MOS-based sensors [36,37,38,39], organic dyes [40], graphene oxide, M-xenes, hydroxylated carbon nanotubes and other nanomaterials [41]. However, only a few studies quantitatively determine the stage of fish spoilage, while others report determining either the late stage of fish spoilage [40] or provide a semi-quantitative classification, such as unspoiled, acceptable, or spoiled [36,42]. This does not allow one to make quantitative predictions about the remaining storage time. To visualize the data obtained from these sensor arrays, principal component analysis or linear discriminant analysis is usually used. However, the decision-making process usually requires more sophisticated approaches, such as support vector machines [36,42] or artificial neural networks [41,43]. A detailed comparison of various approaches is presented in [39].

In this study, we developed, optimized, and applied a new type of the electronic nose to quantitatively identify the freshness of fish. The artificial nose is based on the electrochemical chemotransistor [44], serves as a virtual sensor array providing multiple independent signals from a single sensing element [45]. Significant attention was paid on the optimization of the electrolyte which connects reference electrode with chemosensitive polymer: insufficient stability of this electrolyte was the bottleneck in the former applications of this system. In contrast to other works, we used a simple, transparent data analysis approach based on calculating the Euclidean distance between the measured descriptors and those obtained from the database during sensor calibration. This approach requires only a small dataset for calibration and provides transparency in the analysis.

## 2. Materials and Methods

### 2.1. Sensor Chip Fabrication

An integrated electrochemical transistor based on a five-electrode configuration was used. The 150 nm thick gold electrodes were prepared using the photolithographic lift-off technique on a 0.5 mm thick glass wafer (Fraunhofer IZM-München, Germany, Munich). A 15 nm TiW sublayer was used to improve adhesion of the gold to the glass. This configuration corresponds to that described in [46], but without an auxiliary electrode. The 150 nm thick electrodes were deposited on the glass chip using the photolithographic lift-off technique. The outer electrodes were 10 µm wide, the inner electrodes were 5 µm wide, and the gap between the electrodes was 5 µm. The electrodes were “folded” onto the surface to form a compact 0.4 × 0.4 mm structure (see [47] for a detailed description). Before functionalization, the gold electrodes were cleaned with successive treatments in acetone, ethanol, distilled water, and a piranha solution (a 1:3 mixture of 30% H_2_O_2_ and concentrated H_2_SO_4_, *v*/*v*). Finally, they were thoroughly rinsed with distilled water. Caution: The piranha solution reacts violently with most organic materials and must be handled with extreme care. A silver/silver chloride electrode was formed on the corresponding gold electrode by cycling the silver potential from a solution containing 10 mM AgNO_3_ (Sigma-Aldrich, Germany, Taufkirchen), 20 mM EDTA (Merck, Germany, Darmstadt), 120 mM NH_4_OH (Sigma-Aldrich), and 80 mM NaOH (ROTH), and then the chloride potential from a solution containing 0.1 M HCl (ROTH, Germany, Karlsruhe).

### 2.2. Formation of Chemosensitive Layer

Polyaniline films were prepared via electrochemical synthesis using a PalmSense EmStat4S (Houten, The Netherlands) with an ordinary three-electrode configuration. This configuration contained a saturated Ag/AgCl reference electrode with a salt bridge (saturated KCl) and a platinum wire counter electrode. The synthesis was performed in a 0.5 M H_2_SO_4_ solution containing 0.1 M aniline. Electrochemical synthesis was performed by cycling the electrode potential between +0.9 and −0.2 V at a scan rate of 100 mV/s (Figure S1a,b). During optimization, the polymerization process was stopped when the oxidation peak reached ~200 µA. Subsequent work decreased this value to ~80 µA to produce a smoother polymer film and prevent possible polymer detachment due to the formation of large polymeric grains. Setting this value allowed us to obtain similar polymer thicknesses. After polymerization, the electrodes were rinsed thoroughly with distilled water. Polyaniline films were prepared via electrochemical synthesis using a PalmSense EmStat4S with an ordinary three-electrode configuration containing a saturated Ag/AgCl reference electrode with a salt bridge (sat. KCl) and a platinum wire counter electrode from a 0.5 M H_2_SO_4_ solution containing 0.1 M aniline. Electrochemical synthesis was performed by cycling the electrode potential between +0.9 and −0.2 V at 100 mV/s (Figure S1a,b). During optimization, the polymerization process was stopped when the oxidation peak reached ~200 µA. In subsequent work, this value was decreased to ~80 µA to produce a smoother polymer film and prevent possible polymer detachment due to the formation of large polymeric grains. Fixing this value allowed us to obtain similar polymer thicknesses. After polymerization, the electrodes were rinsed thoroughly with distilled water.

### 2.3. Formation of Electrochemical Chemotransistor

The two- and four-point (s24) simultaneous technique, as previously described in references [47,48], was applied to the in-situ conductivity measurements. The measurement rate was approximately 5 s per data point. During this time, the resistance was calculated based on the difference in current between two opposite voltage pulses. The redox state of the electrode was controlled by applying a direct current (DC) voltage between one of the four measurement electrodes (sense low) and the Ag/AgCl reference electrode integrated into the sensor chip. Details are described in [46]. Then, a thin layer of non-evaporable electrolyte was applied to the sensor chip surface. Seven imidazolium-based ionic liquids containing chloride as the anion were tested as this electrolyte: 1-benzyl-3-methylimidazolium chloride, 1-butyl-3-methylimidazolium chloride, 1-butyl-2,3-dimethylimidazolium chloride, 1-hexyl-3-methylimidazolium chloride, 1-methyl-3-octylimidazolium chloride, 1-allyl-3-methylimidazolium chloride, and 1-methyl-3-octylimidazolium chloride (Sigma-Aldrich). The constant activity of the chloride ion in these media provides a constant potential of the internal Ag/AgCl electrode. In experiments with an integrated reference electrode (electrochemical chemotransistor), the five-electrode configuration (without auxiliary electrode) was used. The thick AgCl layer on the Ag/AgCl reference electrode surface acted as a charge buffer for the chemosensitive material’s redox conversions.

### 2.4. Modification of Ionic Liquid and Chemosensitive Study

The electrolyte was prepared (if another is not indicated) by mixing 70 µL of a 15% solution of poly(2-acrylamido-2-methyl-1-propane-sulfonic acid) (PAMPSA) (from Sigma-Aldrich), 50 µL of 1-decyl-3-methylimidazolium chloride (from Sigma-Aldrich), 60 µL of water, and 5 µL of Triton X-114 (from Sigma-Aldrich). The chemosensitive properties of the electrochemically formed receptor were tested using a 4.2 M trimethylamine (TMA) solution in ethanol (Sigma-Aldrich). 100 µL of TMA were injected into a 100 mL flask at room temperature and incubated for ~30 min to establish a saturated vapor atmosphere. Then, defined volumes of TMA vapor were drawn with a syringe and injected into the sensor chamber for qualitative analysis.

### 2.5. Fish Samples and Storage Conditions

The fish samples were cultured seabream (*Sparus aurata*) and commercially available escolar (*Lepidocybium flavobrunneum*). The fresh fish were filleted into small pieces, approximately 8.5–9.0 cm by 4.5–5.0 cm, and frozen at −20 °C on the day of capture. Immediately before the experiment began, one fillet piece was thawed and cut into 1 g pieces. The pieces were then stored at 5 °C for a defined period of time. To account for biological variability and ensure reproducible results, the study used filleted samples from five fish of the same species. For each storage time interval, measurements were performed at least four times for every sample.

Fish measurements were conducted in a Plexiglas cell. The cell was designed to be airtight and to minimize the free volume between the fish sample and the working part of the sensor chip. Fish measurements were conducted in the Plexiglas cell. The cell’s design ensured tightness and minimal free volume between the fish sample and the working part of the sensor chip. This allowed vapors from the fish to reach saturated pressure.

The temperature value used to define freshness should allow for measurable changes in fish products within a reasonable timeframe. At freezing temperatures, it can take months or even years to observe such changes, which is not suitable for sensor calibration. At room temperature, significant changes occur within the first two days, making the time scale too short and influencing the results. Therefore, a storage temperature of +5 °C was selected as a compromise. This temperature simulates realistic domestic refrigeration conditions and potential minor temperature abuse during retail or transport. Although optimal industrial storage is closer to 0 °C, 5 °C is a more common real-world scenario in which spoilage accelerates. This makes it an ideal resting condition for validating the proposed sensor array. This temperature allowed us to monitor the dynamic generation of volatile amines within a practical experimental timeframe while remaining within realistic chilled storage conditions.

Storing fish samples at this temperature led to the formation of a characteristic spoilage odor within four days. Therefore, it is reasonable to conduct an investigation within eight days of catching the fish or thawing frozen samples.

## 3. Results and Discussion

### 3.1. Selection of Ionic Liquid

**Figure 1:**
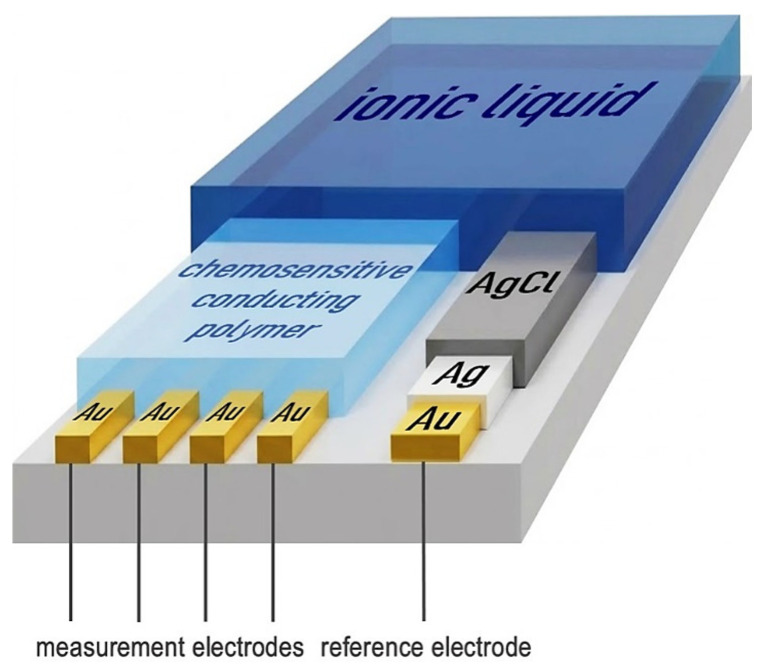
The design of the electrochemical chemotransistor. Four chemosensitive conducting polymer-covered measurement electrodes perform simultaneous two- and four-point measurements [47,48]. The fifth electrode being coated by Ag/AgCl operates as the reference electrode and as the electrode controlling the redox-state of the chemosensitive conducting polymer.

The first studies on electrochemical chemotransistors [46] used a water-based gel electrolyte to connect the measurement and control electrodes for the redox state of the chemosensitive polymer (Figure 1). However, evaporation of the water from the gel electrolyte caused changes in the chloride concentration. This first led to potential drift of the silver/silver chloride reference electrode, and later to breakdown of the electrical connection and disintegration of the system. Using a chloride-containing ionic liquid instead of a gel electrolyte improved the chemotransistor’s operating time [49]. Compared to a water-based gel electrolyte, chloride-containing ionic liquids have several advantages: (i) they have very low vapor pressure, so the evaporation rate is much lower and the sensor’s operating time is much longer, (ii) most ionic liquids have a lower surface tension than water. Therefore, the diffusion of gases through the spread layers is faster, and the layers are thinner, (iii) the chloride concentration in chloride-based ionic liquids is much higher than the solubility limit of most chloride-containing salts in water. This provides better stability of chloride activity near the integrated silver/silver chloride reference electrode and decreases potential drift.

In this study, we compared chloride-containing ionic liquids for use in electrochemical chemotransistors. The ionic liquids considered are shown in Table 1. The following criteria were applied to the comparison: (i) minimal evaporation rate, and (ii) minimal water accumulation from the surrounding air. The first criterion characterizes sensor lifetime. The second criterion characterizes potential stability; water accumulation decreases chloride activity and shifts the reference electrode potential. Furthermore, significant water accumulation can increase analyte diffusion time.

**Figure 2:**
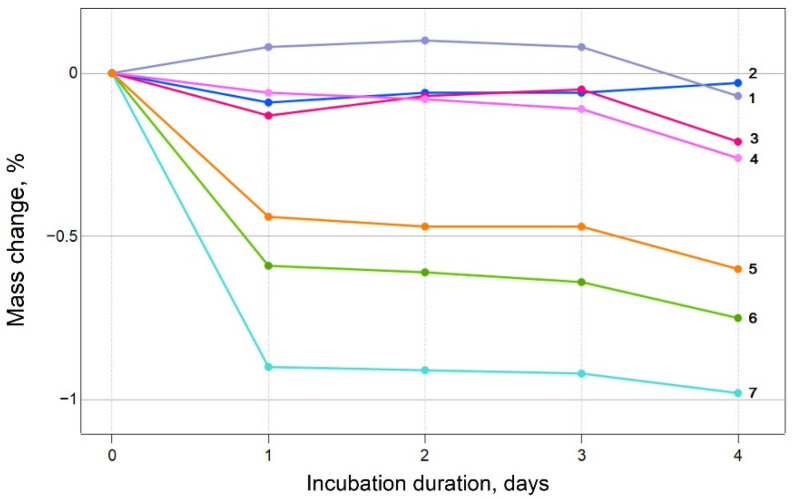
Change in the mass of chloride-based ionic liquids during incubation at 110 °C. Ionic liquids: **1**—1-butyl-2,3-dimethylimidazolium (lilac curve), **2**—1-allyl-3-methylimidazolium (blue curve), **3**—1-butyl-3-methylimidazolium (magenta curve), **4**—1-hexyl-3-methylimidazolium (light magenta curve), **5**—1-benzyl-methylimidazolium (orange curve), **6**—1-methyl-3-octylimidazolium(green curve), **7**—1-decyl-3-methylimidazolium (cyan curve).

An evaporation investigation was performed using thermogravimetric analysis while ionic liquids were incubated in 2000 µL aluminum cans without cups at 110 °C. Initial experiments showed that primary thermal exposure of the samples for 96 h at 60 °C is necessary for reproducible results. This may be due to impurities of low molecular weight. After this pretreatment, the mass loss over the course of a 96 h incubation period at 110 °C was examined (Figure 2). The results revealed that the ionic liquids 1-benzyl-methylimidazolium, 1-methyl-3-octylimidazolium, and 1-decyl-3-methylimidazolium lost 0.6–1% of their mass. Meanwhile, the mass loss for 1-butyl-2,3-dimethylimidazolium, 1-allyl-3-methylimidazolium, 1-butyl-3-methylimidazolium, and 1-hexyl-3-methylimidazolium was below 0.25%. Extrapolating to room temperature, based on typical activation energy values gives an evaporation rate below 0.4% per year for all the compounds studied. Such evaporation and the corresponding decrease in layer thickness are certainly not essential to sensor degradation. Notably, the main mass loss occurred during the first day of incubation (Figure 2), suggesting that the actual mass loss during long incubations is much lower than estimated above. Therefore, all of the studied ionic liquids fulfill the requirements of the first criterion.

**Figure 3:**
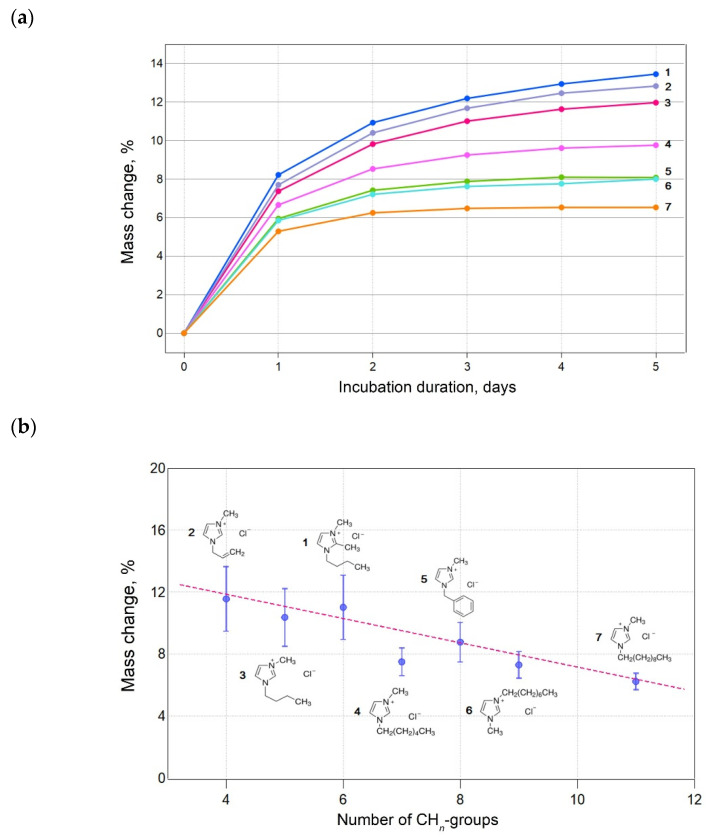
Accumulation of water from air with 75% humidity at room temperature in various ionic liquids (**a**): **1**—1-butyl-2,3-dimethylimidazolium (lilac), **2**—1-allyl-3-methylimidazolium (blue), **3**—1-butyl-3-methylimidazolium (magenta), **4**—1-hexyl-3-methylimidazolium (light magenta), **5**—1-benzyl-methylimidazolium (orange), **6**—1-methyl-3-octylimidazolium(green), **7**—1-decyl-3-methylimidazolium (cyan) and a correlation of mass increase due to water absorption with the hydrophobicity of ionic liquids, estimated as the number of (CH)*_n_*-groups in the molecule (**b**). The line corresponds to the linear approximation.

The investigation of water accumulation involved a five-day incubation in air with 75% humidity at a temperature of ~20 °C. The results are shown in Figure 3a. The mass increase varied significantly among the compounds. The highest mass increase rate occurred at the beginning of the incubation period, gradually decreasing with a trend toward saturation. This can be explained by a decrease in the gradient of the chemical potential of water between the two phases (air and ionic liquid), which results in an accumulation of water in the ionic liquid. The best results were obtained for 1-decyl-3-methylimidazolium; the water accumulation stopped by the second or third day, reaching 6.5%. Similar results were obtained for 1-methyl-3-octylimidazolium and 1-methyl-3-octylimidazolium; however, the total mass increase was approximately 8%. The data obtained on water accumulation correlates well with the hydrophobicity of ionic liquids. This dependence is shown in Figure 3b. The “hydrophobicity index” was calculated based on the number of CH*_n_* groups (*n* = 1, 2, or 3) in the molecule. An accumulation of 7% water (and the corresponding decrease in chloride activity) leads to a potential change in the silver/silver chloride electrode of less than 2 mV. This change is not significant for fixing the redox state of the chemosensitive polymer.

Based on these data, 1-decyl-3-methylimidazolium chloride (DMICl) was selected as the most suitable ionic liquid for electrochemical chemotransistors. Electrochemical studies demonstrated that DMICl is electrochemically stable within a potential window of −2 to +2 V versus Ag/AgCl in this medium (Figure S2).

### 3.2. Optimization of Electrolyte

**Figure 4:**
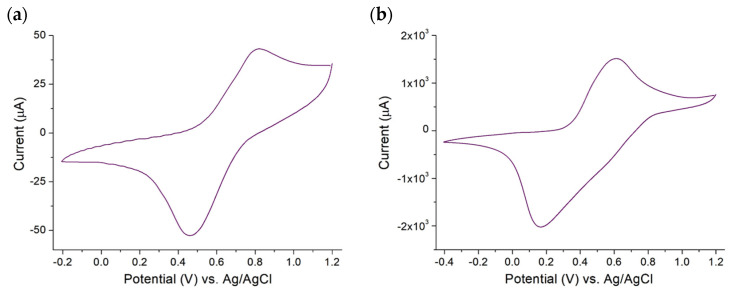
Electrochemical activity of PANI in pure 1-decyl-3-methylimidazolium (**a**) and in the mixture of 1% (*w*/*v*) PAA and 1-decyl-3-methylimidazolium (**b**) measured with macroscopic wire electrodes. Scan rate: 50 mV/s.

**Figure 5:**
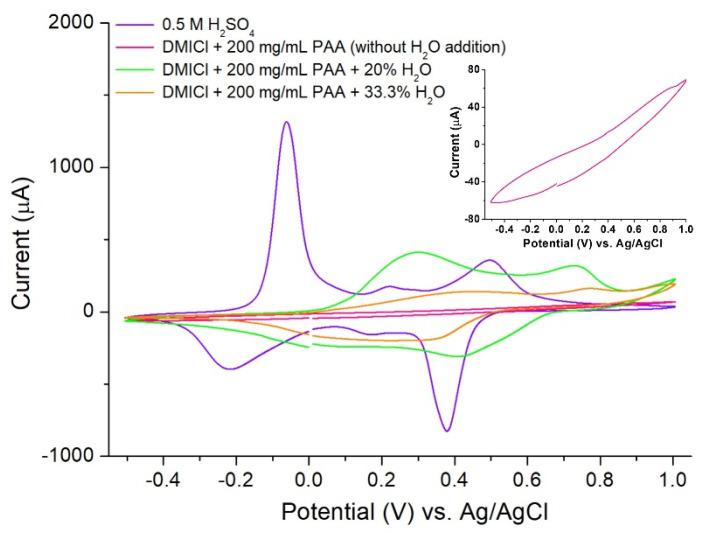
Cyclic voltammograms of PANI in 1-decyl-3-methylimidazolium containing PAA in non-aqueous conditions (the same in the inset, magnified) and after addition of 20% and 33% of water. For comparison, the results measured in 0.5 M sulfuric acid are shown.

The electrochemical activity of the chemosensitive polymer in pure ionic liquid is very low (Figure 4a). However, adding an acidic dopant that provides protons for the polymeric aniline (PANI) to convert into its conductive emeraldine salt form significantly increases this activity. After an initial study of acidic dopants, including phosphoric acid, citric acid, and lactic acid, it became clear that only polymeric acidic compounds could provide the necessary stability. Thus, detailed testing was performed with two polymeric acids: polyacrylic acid (PAA), which has a pKa of ~5.8 [61], and poly(2-acrylamido-2-methyl-1-propane sulfonic acid) (PAMPSA), which has a pKa of ~1.5 [62]. Figure 4 shows the results of adding a 1% (*w*/*v*) aqueous solution of PAA (MW 400 kD) at a 1:1 (*v*/*v*) ratio to the ionic liquid. The electrochemical activity of PANI, which is pronounced in an aqueous sulfuric acid solution, is blocked in a pure ionic liquid (Figure 4a and Figure 5). Adding PAA increased the oxidation/reduction peaks from 40–60 µA to 1.5–2.0 mA (Figure 4b), as measured with a macroscopic wire electrode. A similar effect was observed with chemotransistor electrodes.

A dramatic increase in the electrochemical activity of PANI was also observed after the addition of an acidic dopant with other ionic liquids, such as 1-hexyl-3-methylimidazolium and 1-methyl-3-octylimidazolium (Figure S3). Increasing the PAA concentration from 1% to 25% (*w*/*v*) led to a gradual increase in electrochemical activity and the formation of more pronounced oxidation and reduction peaks of PANI. However, due to lower evaporation and water absorption, 1-decyl-3-methylimidazolium was only used as the basis for the electrolyte in further experiments.

**Figure 6:**
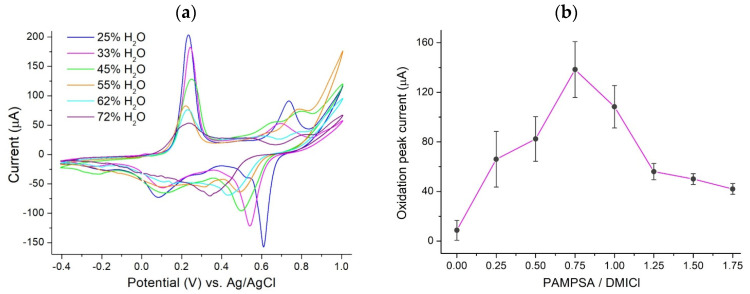
Influence of water (**a**) and PAMPSA (**b**) additives into ionic liquid (DMICl) on the electrochemical activity of polyaniline. Cyclic voltammogramms of polyaniline for various water content in ionic liquid; scan rate: 50 mV/s vs. Ag/AgCl (**a**). Dependence of the first oxidation peak current of polyaniline on the PAMSA/DMICl ratio (*v*/*v*), measured in the presence of 33% at scan rate of 50 mV/s vs. Ag/AgCl (**b**).

To consider the possible influence of water content, the electrochemical activity of PANI was tested under non-aqueous conditions. First PAA was introduced into 1-decyl-3-methylimidazolium in powder form. Then, a small amount of water was added (Figure 5). There was no electrochemical activity of PANI under non-aqueous conditions. Adding a small amount of water caused the formation of PANI redox peaks. However, the observed electrochemical activity was much lower than in an aqueous sulfuric acid solution (Figure 5). The shift in peak potentials may be partially due to the chloride ion activity difference between the sulfuric acid experiment (salt bridge with saturated KCl in water) and the ionic liquid experiment, as this activity defines the potential of the reference electrode. Notably, the effect of water on the observed electrochemical activity of PANI is non-monotonous. Increasing the water concentration from 20% to 33% decreased the activity. Introducing PAMPSA into the ionic liquid produced more pronounced electrochemical activity (Figure 6). This can be explained by its lower pKa. PAMPSA was introduced into the ionic liquid as a 7% aqueous solution at a ratio of 1:1.33 (*v*/*v*). The results were compared with measurements in 0.5 M sulfuric acid (Figure S4), and very similar cyclic voltammograms were obtained (Figure 6a). The observed shift in peak positions is close to the measured potential difference between the Ag/AgCl (sat.) electrode with a salt bridge and the Ag/AgCl electrode in the ionic liquid (~280 mV). However, a quantitative comparison of the potential shift is complicated by the unknown contribution of the diffusion potential at the salt bridge/ionic liquid interface. Further optimization was performed by introducing additional water to vary its concentration between 10% and 50%. The magnitude of the oxidation peak, which corresponds to the leucoemeraldine-to-emeraldine salt transition [63,64], was used to measure electrochemical activity. The formulation containing 33.3% water exhibited the highest oxidation current, over 300 µA (Figure S5). Increasing the water content to 72% further decreased the separation of the oxidation and reduction peaks, as well as their magnitude, and increased their width. Based on these results, a water content of 33.3% was selected for further study.

The next step was to determine the optimal PAMPSA ratio. Using the same electrochemical activity criterion, we varied the PAMPSA-to-ionic liquid ratio (*v*/*v*) from 0 to 1.75/1 (see Figure 6b and Figure S6). Under H^+^-shortage conditions (ratios below 0.75), the oxidation current increased sharply from approximately 10 µA to 140 µA, indicating progressive improvement in ionic transport and charge-transfer kinetics. The maximum oxidation current was reached at an ionic liquid-to-acid ratio of 3/4, indicating an optimal composition that balances acidity and ionic mobility. Beyond this value, however, the oxidation current decreased progressively, falling to approximately 40 µA at a ratio of 7/4 (Figure 6b). A similar dependence was observed in the resistance measurements (Figure S6). For these data, the dependence on PAMPSA content was also non-monotonous, with minimal values in the middle of the investigated range. Notably, the ratio of the two- and four-point resistances was ~3 for most of the measured ratios, indicating low contact resistance contribution and good polymer adhesion in such an environment.

The final formulation, consisting of 1-decyl-3-methylimidazolium, PAMPSA, and water, was used as the optimal electrolyte to connect the measurement and reference electrodes of electrochemical transistors. Later steps of its application included the addition of 1% non-ionic surfactant Triton X-120 (*v*/*v*). This was done to reduce the surface tension, which was too high and led to the formation of a thick liquid layer on the sensor surface. This resulted in correspondingly slow analyte diffusion through this layer. Introducing a small amount of non-ionic surfactant does not affect the system’s electrochemical properties, but it decreases the electrolyte’s surface tension and improves its spreading. This results in the formation of thin layers.

**Figure 7:**
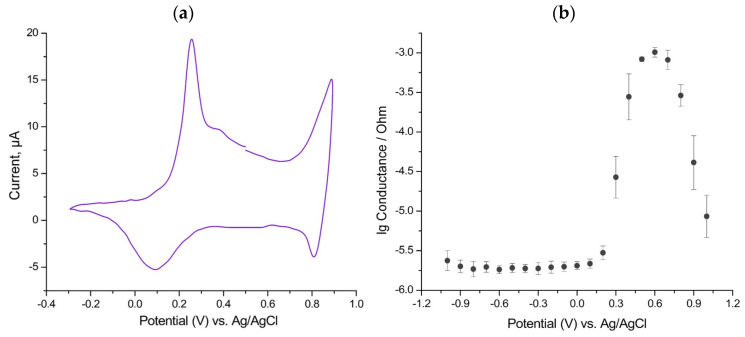
Cyclic voltammetry (**a**) and 4-point conductivity (**b**) of the optimized chemotransistor. Connecting electrolyte: mixture of 1-decyl-3-methylimidazolium, PAMPSA, water, and Triton X120. Scan rate for (**a**): 100 mV/s.

Figure 7a shows the cyclic voltammetry of the final sensor. It corresponds to the well-known cyclic voltammetric curves of PANI and exhibits two redox states: one at potentials below 0.1 V (suggestively, leucoemeraldine) and one between 0.4 and 0.8 V (suggestively, emeraldine base). The second peak at ~0.85 V indicates a transition to the next oxidation state, which was not achieved within the potential range used. Similar transitions can be observed in the conductivity curve (Figure 6b): the first redox state below 0.1 V, the second redox state around 0.6 V, and an incomplete transfer to the next oxidation state. These data allow us to select the corresponding potentials for measurements in the virtual sensor array configuration.

### 3.3. Measurements of Trimethylamine

Since trimethylamine is a primary component of the headspace of fish, the sensor’s response to this compound was studied. The response kinetics corresponded to a monoexponential dependence (Figure 7a). This is often the case for various affinity sensors and indicates transfer kinetics between two energetic states. However, this does not rule out diffusion control, so using kinetic parameters to calculate binding constants is questionable. Earlier work with a different electrolyte composition in an electrochemical chemotransistor revealed that the conductance kinetics upon trimethylamine injection also obeyed a monoexponential function [44]. Furthermore, using a complex, multicomponent analyte (fish headspace) leads to more complex, non-exponential kinetics.

**Figure 8:**
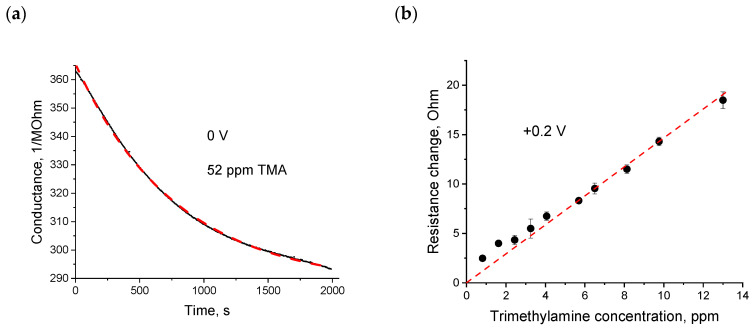
Sensor response on addition of trimethylamine: an example of response kinetics (**a**) and concentration dependencies of the resistance changes (**b**). The kinetics (black) was fitted (red) by monoexponential function. More examples are shown in Figure S6.

The response magnitude was monotonically dependent on analyte concentration (Figure 8 and Figure S7). The dependence was nearly linear for resistance (Figure 8b). However, the dependence for the conductance change obeyed the Langmuir adsorption isotherm, with binding constants ranging from 20 to 70 ppm. This confirms the model of the sensitive polymer as a circuit of parallel resistors [65]. According to the IUPAC definition, the analytical sensitivity was calculated as the slope of the two-point resistance change for electrode voltages of −0.8 V, 0 V, and +0.6 V, estimated as 3000 kΩ/ppm, 30 kΩ/ppm, and 0.25 kΩ/ppm, respectively. For four-point resistance, these values were approximately three to four times lower, indicating that the measured resistance is that of the polymer and that the contribution of contact resistance is very low [47,48]. The detection limit was below 1 ppm. Data analysis for such a simple analyte in the sensor application can be performed conventionally using the measured concentration dependencies as the calibration curve.

### 3.4. Measurements of Fish Headspace

#### 3.4.1. Data Analysis

For the analysis of fish freshness, an electrochemical transistor was used in a virtual sensor array configuration. Measurements were performed at various potentials corresponding to different redox states of the chemosensitive material. These data were then used to reach the final conclusion.

**Figure 9:**
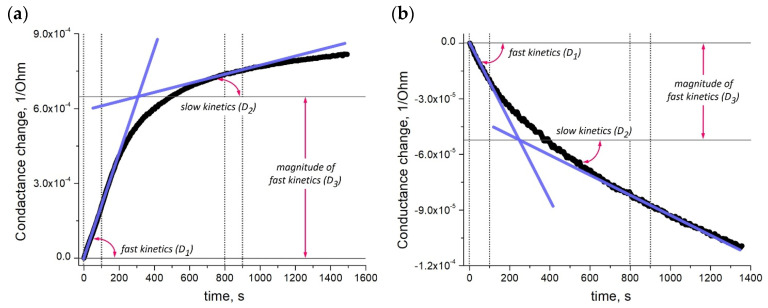
Changes in the sensor signal in the measurement cell containing a fish sample on the fourth day of storage at potentials of: +0.3 V (**a**) and +0.6 V (**b**). Electrolyte composition: 1-decyl-3-methylimidazolium chloride, poly(2-acrylamido-2-methyl-1-propane sulfonic acid), and Triton X-114. The fish sample is cultured seabream (*Sparus aurata*). The lines indicate two extracted descriptors, and the third descriptor is obtained by crossing these lines.

The interaction between the sensor and the fish headspace resulted in changes to the sensor’s conductance (Figure 9). The kinetics and response sign of the sensor responses varied depending on the redox state of the chemosensitive material and the storage time of the sample. At the same time, the obtained kinetics were complex and not exponential. This is certainly due to the presence of multiple different compounds in the fish headspace. These compounds have various origins and possess different kinetic and affinity properties, as well as different effects on sensor conductivity. Even if each compound exhibited exponential dependence, the sum of exponents would not yield an exponential function. The complex kinetics required an empirical approach to extract a set of descriptors from the complex sensor response for further data analysis.

Different approaches were tested to find these descriptors. The use of biexponential function to fit obtained kinetics was not optimal: the slow parts of the sensor responses had predominantly linear character; this which leads to an undefined characteristic time of the slow exponent. Finally, the following 3 descriptors were selected for each kinetic response of the sensor:

▪ ***D*_1_** as the rate of the fast kinetics, obtained by linear regression for the first 100 s,
▪ ***D*_2_** as the rate of the slow kinetics, obtained by linear regression for the time from 800 s to 900 s, and
▪ ***D*_3_** as the magnitude of the fast kinetics defined by the intersection of the regression lines obtained for the calculation of ***D*_1_** and ***D*_2_** (Figure 9a,b).

The descriptors characterize the observed two-stage, non-exponential kinetics. The descriptors ***D*_1_** and ***D*_2_** characterize the fast and slow process rates, respectively, while ***D*_3_** characterizes the contribution of the fast process. The descriptors were normalized to the standard deviation for all storage durations. Of course, these three descriptors do not provide a complete description of the measured signals; one cannot reconstruct the signal shape based on them. However, as will be shown below, they contain enough information to analyze fish freshness.

**Figure 10:**
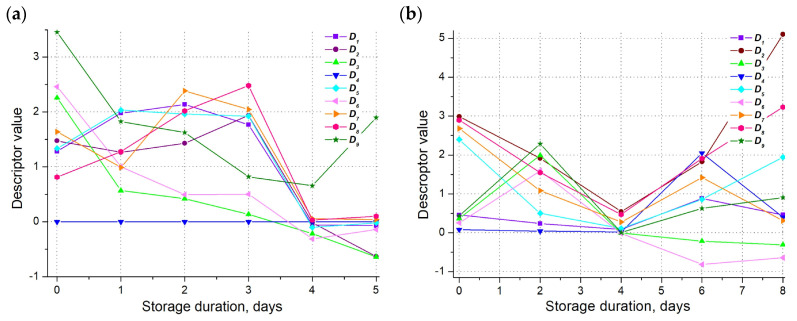
The dependence of the extracted descriptors on storage time. The data correspond to the Table S1.

These data were collected for different redox states of the chemosensitive material, which are defined by electrode potential. Three electrode potential values were usually used, leading to the extraction of nine total descriptors for each storage day of the fish sample. The measured values were normalized according to the variation in the corresponding value across all measurements. This normalization allows us to compare different descriptors as dimensionless values of a similar order. Examples of the extracted descriptors are shown in Table S1. Correlation analysis of most pairs of the nine descriptors demonstrated relatively low mutual correlations (Table S2). The mean square values, excluding diagonal elements, are 0.52 and 0.62, respectively. Thus, using a combination of these descriptors increases the total amount of information on the studied object. The extracted descriptors exhibit complex, non-monotonous dependencies on storage time (Figure 10a,b). Therefore, simple regression-based analyses are not applicable.

#### 3.4.2. Mathematical Evaluation of the Equivalent Storage Time

The extracted descriptors can be presented as characteristic patterns (Figure S7) for different storage times. Determining the most probable storage time means recognizing the experimentally obtained descriptor pattern by comparing it with the patterns indicated in Figure S7. Pattern recognition is a well-studied field of mathematics, and many sophisticated approaches have been developed for this purpose. However, our goal was to use a simple method that allows us to work with a small dataset and provides a transparent decision-making process. In principle, this task can be solved by maximizing mutual correlations. However, we used an even simpler approach that is often used in data clustering.

The most probable equivalent storage time value for fish samples was determined by minimizing the Euclidean distance between the measured descriptors and the mean values of the descriptors measured during the calibration procedure for different storage durations. This distance was calculated in the multidimensional space formed by the descriptor basis. Due to the low mutual correlations between the descriptors, this basis can be considered orthogonal. Under this assumption, the Euclidean distance (***d***) between the measured values of the descriptors, ***D_i,x_*** (*i* = 1, …, 9), for a sample with storage time *x*, and the mean values of these descriptors, ⟨***D_i_***⟩, can be calculated as follows:

$$\delta_{k}\;=\;\sqrt{\sum_{i=1}^{N}{\left(D_{i,x}-\left\langle D_{i,k}\right\rangle \right)}^{2}},$$

where ***D_i_*** is the measured value of descriptor ***i***, ⟨***D_i,k_***⟩ is the mean value of descriptor ***D_i_*** for storage time ***k***, and ***N*** is the total number of descriptors in each set for storage time ***k*** (in our work, ***N*** = 9). Figure 10 illustrates an application of this approach to a simplified case in which only two descriptors (***D*_1_** and ***D*_2_**) are used. A comparison of the Euclidean distances ***δ_k_*** allows us to associate an unknown descriptor set with the closest value of parameter ***k*** (Figure S9).

**Figure 11:**
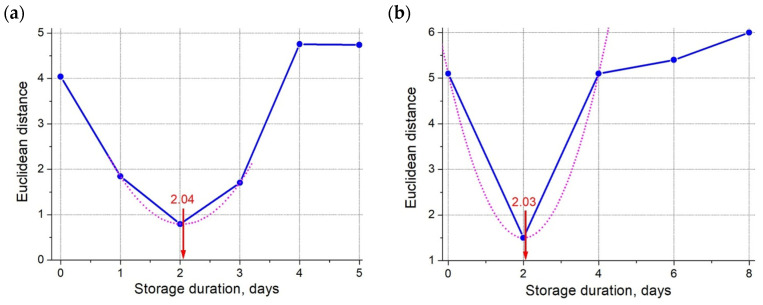
Euclidean distances for fish samples stored for two days. The minimum values correspond to two days. The magenta curves show the parabolic function fitted to the three points around the minimum; the minimum values of this fit are shown by the arrows. The samples are: *Lepidocybium flavobrunneum* (**a**) and *Sparus aurata* (**b**).

Figure 11 illustrates the application of this approach to determining the storage time of fish samples. In this example, the storage time of the fish samples was two days, which corresponds to ***k*** = 2 (***k*** = 0 corresponds to fresh samples). The comparison shows that ***k*** = 2 corresponds to the minimum Euclidean distance between the measured normalized descriptors and the corresponding values in the database.

The lower parts of the curves in Figure 11a,b can be approximated by a parabolic curve to make a more exact determination of the minima corresponding to storage time. In this case, the width of the curve characterizes the quality of the analysis, and the minima of the curves can be considered a more precise determination of the storage time. While this approach may be attractive to end users, it requires voluntary decisions to select the fitting function, so it does not have a strong mathematical and statistical basis. Nevertheless, this approach leads to reasonable results. The dotted red curves show the fitting of three points with the lowest Euclidean distances by a parabolic function; the minima of the functions are shown by arrows. The corresponding storage time values are 2.04 days (Figure 11a) and 2.03 days (Figure 11b). At the current stage of the study, we cannot say if this “improvement of the precision” does describe the reality or should be considered as an overinterpretation of the data, therefore we round these values till integer number of storage days. Similar tests performed on samples with other storage durations also showed exact agreement with the actual storage time. Although no deviations were observed within the limited data set used here, a rigorous metrological assessment will require a substantial expansion of the dataset. In the same time, we have demonstrated that the selected descriptors of the response of optimized chemotransistors together with a straightforward analysis based on minimizing the Euclidean distance enable successful determination of the above defined quantitative value of the fish freshness.

## 4. Conclusions

According to an FAO estimate [66], 35% of all caught fish are lost due to spoilage or rejection. The rate of fish spoilage depends on fundamental changes determined by the product’s composition and storage conditions. Knowing the remaining storage duration of raw fish enables one to optimize the realization strategy and minimize losses. The approach developed in the current work can achieve this. By introducing standard storage conditions, we formalized the definition of freshness, making it a measurable quantity. Furthermore, we developed an approach to determine the freshness of fish samples quantitatively based on the defined freshness.

For the freshness measurements, we have applied the recently developed virtual sensor array based on electrochemical chemotransistors [48,67]. Simultaneous 2- and 4-point resistance measurements provided internal control of sensor integrity, which is important for reliable sensor applications. Previously, this system was used to accelerate sensor recovery, extend the concentration range of the glucose sensor, modulate affinity, and form a virtual sensor array. Here, we optimized the system and demonstrated its application to analyze fish freshness. The complex response kinetics and non-monotonous response behavior over time make it impossible to apply regression-based approaches directly to analyze the data. In principle, it would be possible to use neural networks for this purpose. However, this would require a significantly larger amount of data for calibration (network learning). We solved this problem by minimizing the Euclidean distance of the introduced sensor response descriptors. This approach can certainly be applied to many other chemical analytics tasks.

The developed sensor can be considered a laboratory prototype for future industrial applications. At the same time, it is important to note that the sensor is based on the biochemistry of specific fish species. Therefore, applying it to new species requires extracting the corresponding set of descriptors.

## Figures and Tables

**Table 1:** Chloride-containing imidazole-based ionic liquids studied as a possible electrolyte for the electrical connection of the reference and measurement electrodes in electrochemical chemotransistors.

Chemical Name/ Number of Ionic Liquid	Structure	Molecular Weight	Surface Tension	Melting Point
(1) 1-butyl-2,3-dimethylimidazolium	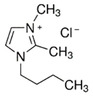	188.7 g/mol	35–45 mN/m [50]	~99 °C [51,52]
(2) 1-allyl-3-methylimidazolium	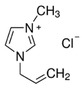	158.6 g/mol	35–53 mN/m [53]	17–19 °C [51]
(3) 1-butyl-3-methylimidazolium	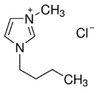	174.7 g/mol	35–45 mN/m [54,55]	~65 °C [51,56]
(4) 1-hexyl-3-methylimidazolium	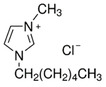	202.7 g/mol	32–46 mN/m [52]	~−2 °C [51]
(5) 1-benzyl-methylimidazolium	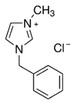	208.7 g/mol	45–55 mN/m [57]	~70 °C [51]
(6) 1-methyl-3-octylimidazolium	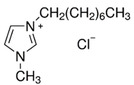	230.8 g/mol	31–43 mN/m [50,58]	~−32 °C [51]
(7) 1-decyl-3-methylimidazolium	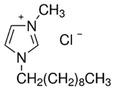	258.8 g/mol	30–42 mN/m [50,59,60]	~38 °C [52]

## Data Availability

The data are available from the authors by request.

## Supplementary Materials

The following supporting information can be downloaded at: https://www.mdpi.com/article/10.3390/s26134306/s1, Figure S1: The electrochemical deposition of polyaniline; Figure S2: The electrochemical stability window of DMICl; Figure S3: Cyclic voltammograms of PANI in [OMIM]Cl (a) and [HMIM]Cl (b) containing stepwise increased concentrations of PAA; Figure S4: Cyclic voltammetry of PANI in 1-decyl-3-methylimidazolium containing 10% wt. PAMPSA and in 0.5 M sulfuric acid; Figure S5: Influence of water and PAMPSA additives into ionic liquid (DMICl) on the electrochemical activity of PANI; Figure S6: Sensor response on addition of trimethylamine: an example of response kinetics and concentration dependencies of conductance decrease; Figure S7: The patterns formed by descriptors for *Lepidocybium flavobrunneum* (a) and for *Sparus aurata* (b); Figure S8. An illustration of minimization of Euclidean distances in the two-dimensional space formed by two descriptors. Table S1: Descriptors and standard deviations; Table S2: Mutual correlations of extracted descriptors.
