# Supplementary material for: A Sensor to Analyze Fish Freshness: A Virtual Sensor Array Based on an Electrochemical Chemotransistor
Source: Sensors (Basel). 2026 Jul 7;26(13):4306. doi: 10.3390/s26134306 (PMC13363832; doi:10.3390/s26134306)
Supplement: Supplementary file 1 [file sensors-26-04306-s001.zip › sensors-4339655-supplementary.pdf]

A sensor to analyze fish freshness: A virtual sensor array based on an electrochemical chemotransistor

Yulia Efremenko[1], Eya Boughanmi[1,2], Vladimir M. Mirsky[1]

[1]Nanobiotechnology Department, Institute of Biotechnology, Brandenburg University of technology Cottbus-Senftenberg, 01968 Senftenberg, Germany; iullia.efremenko@b-tu.de (Y.E.); eya.boughanmi@etudiant-fst.utm.tn (E.B.)

[2]Analytical Chemistry and Electrochemistry Lab, Faculty of Science, University of Tunis El Manar, 2092, Tunis El Manar, Tunisia.

*Corresponding author. E-mail: mirsky@b-tu.de (V. M. Mirsky)

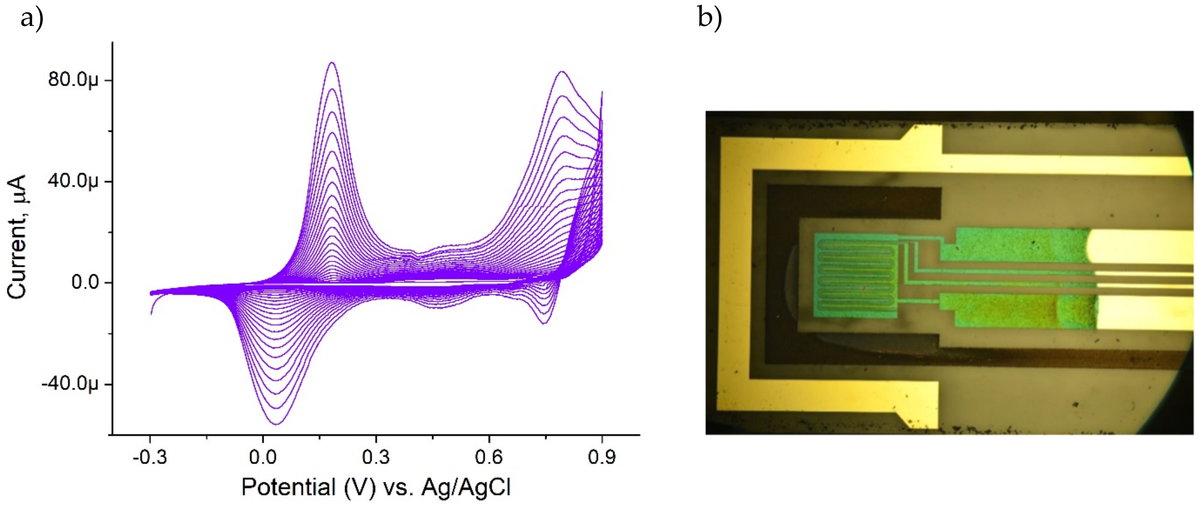

Figure S1. The electrochemical deposition of polyaniline: electropolymerization on the gold electrode within a potential range from -0.2 V to +0.9 V (vs. Ag/AgCl); scan rate: 100 mV/s (a) and microscopic photo of the integrated electrode covered with Ag/AgCl (surrounding strip) and polyaniline (working electrodes for two- and four-point measurements) (b).

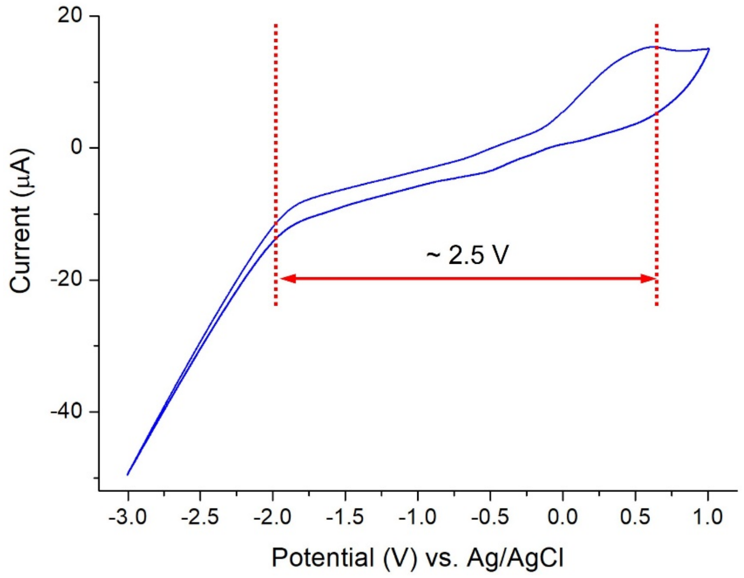

Figure S2. Electrochemical stability window of 1-decyl-3-methylimidazolium chloride vs. Ag/AgCl (salt bridge with sat. KCl) pseudoreference electrode; scan rate: 100mV/s.

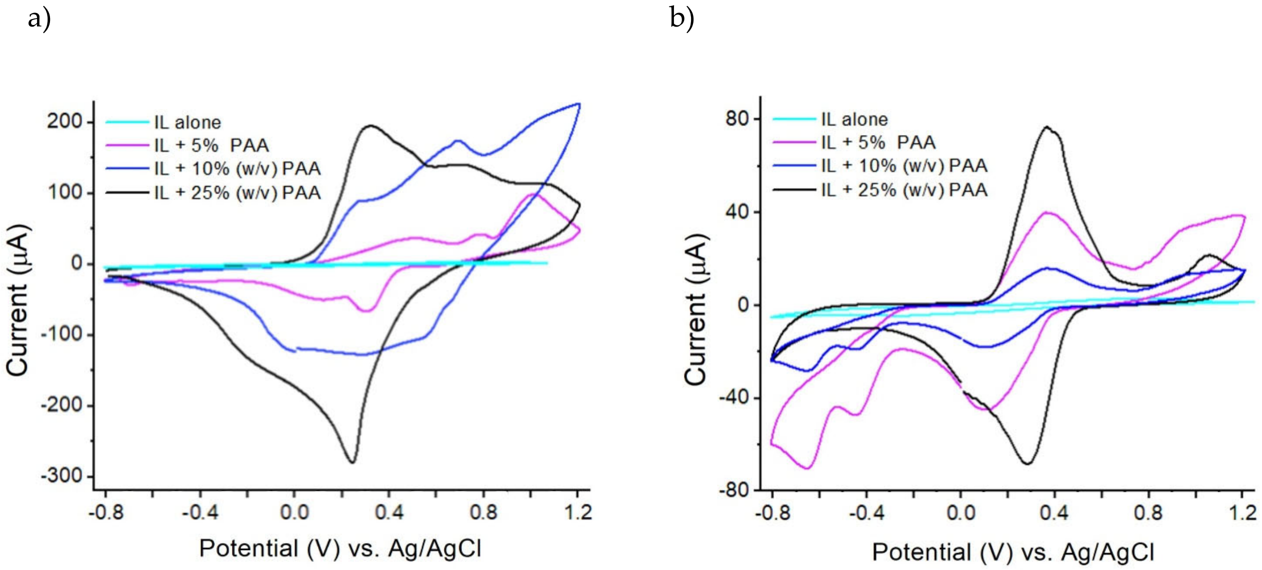

Figure S3. Cyclic voltammograms of polyaniline in 1-methyl-3-octylimidazolium chloride (a) and 1-hexyl-3-methylimidazolium chloride (b) containing stepwise increased concentrations of polyacrylic acid (PAA). The measurements were performed with an integrated Ag/AgCl reference electrode of electrochemical chemotransistor.

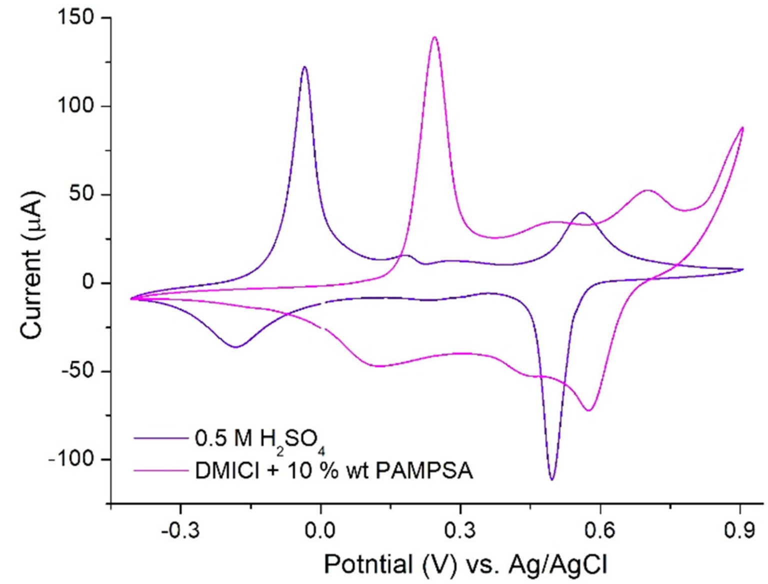

Figure S4. Cyclic voltammetry of polyaniline in 1-decyl-3-methylimidazolium containing 10% wt. poly(2-acrylamido-2-methyl-1-propane-sulfonic acid) (PAMPSA) and in 0.5 M sulfuric acid. Scan rate: 50mV/s. Reference electrode: integrated electrode of electrochemical chemotransistor for measurements in ionic liquid or external Ag/AgCl (sat.) electrode for measurements in sulfuric acid.

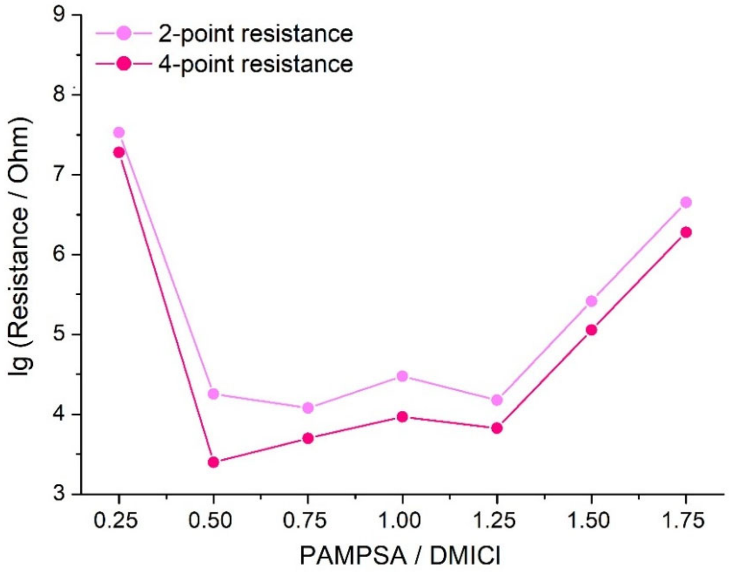

Figure S5. Influence of water and poly(2-acrylamido-2-methyl-1-propane-sulfonic acid) additives into ionic liquid (1-decyl-3-methylimidazolium chloride) on the electrochemical activity of polyaniline: 2- and 4-point resistances of the electrochemical transistor for different content of poly(2-acrylamido-2-methyl-1-propane-sulfonic acid). Electrode potential: 0 V. Reference electrode: integrated electrode of electrochemical chemotransistor.

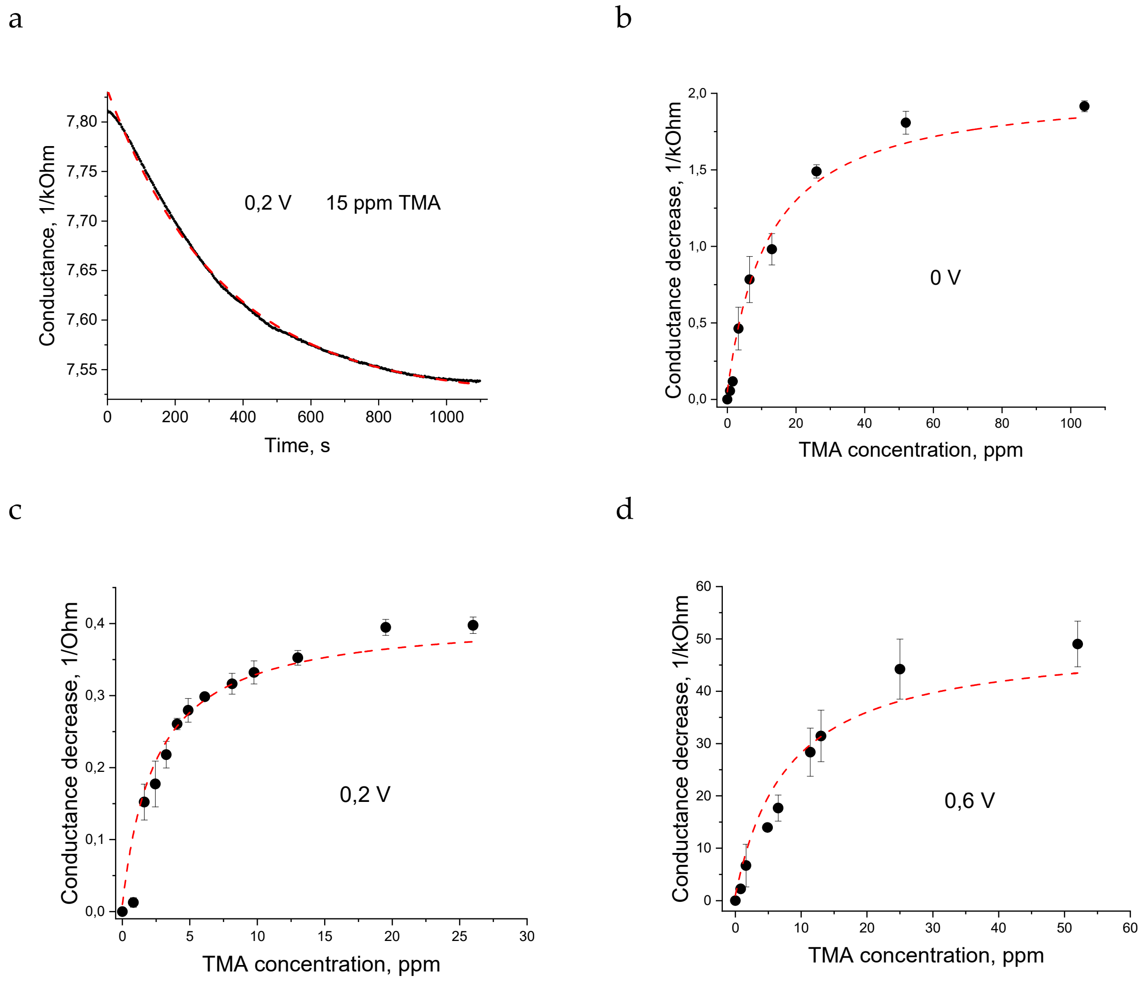

Figure S6. Sensor response on addition of trimethylamine (TMA): an example of response kinetics (a) and concentration dependencies of conductance decrease (c-d). Experimental data (black) are fitted (red) by monoexponential function (a) or by Langmuir isotherm (b-d).

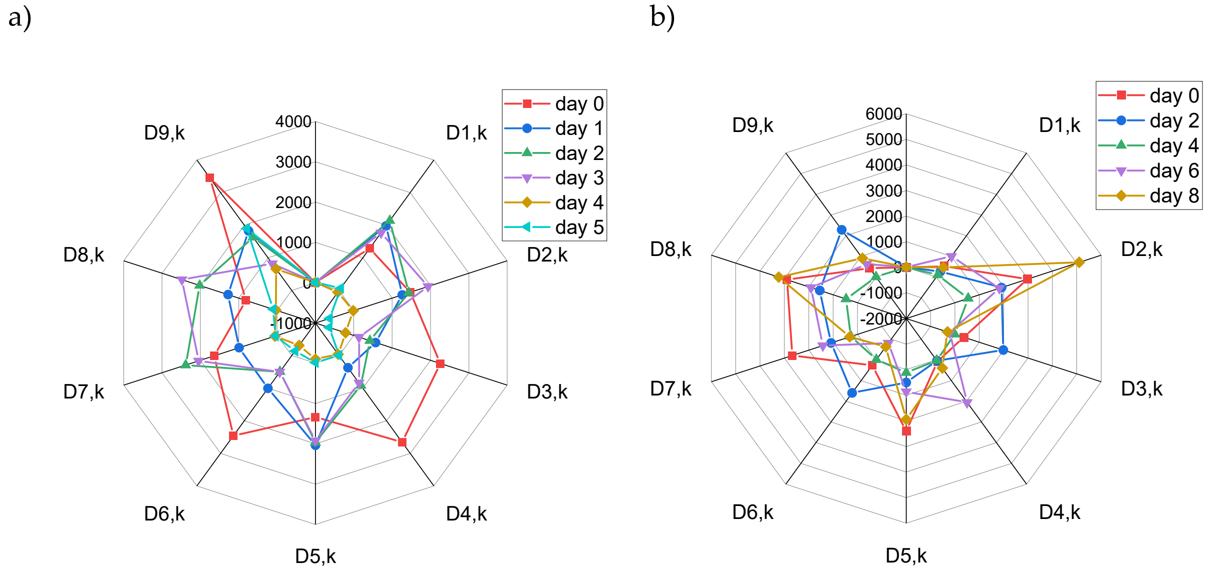

Figure S7. The patterns formed by descriptors for *Lepidocybium flavobrunneum* (a) and for *Sparus aurata* (b).

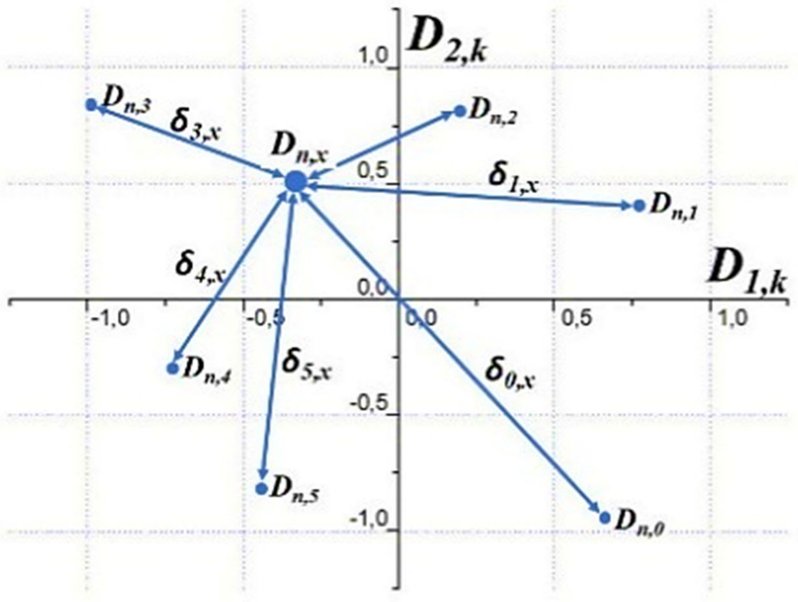

Figure S8. An illustration of minimization of Euclidean distances in the two-dimensional space formed by two descriptors. Here $D_{1,k}$ and $D_{2,k}$ are the coordinates of the first and second descriptor correspondingly, $D_{n,x}$ is the set of descriptors obtained for the measurement of unknown sample x, $D_{0,x}$, $D_{1,x}$, $D_{2,x}$, $D_{3,x}$, $D_{4,x}$, … $D_{9,x}$ are the sets of descriptors for corresponding day of storage from the calibration, and $d_{0,x}$, $d_{1,x}$, $d_{2,x}$, …, $d_{9,x}$ are the Euclidean distances between $D_{n,x}$ and the descriptors from the calibration corresponding to the storage duration k. Minimization of this distance determines the most probable equivalent storage duration of the sample x.

Table S1. Descriptors and standard deviations

k	$D_{1,k}$	$D_{2,k}$	$D_{3,k}$	$D_{4,k}$	$D_{5,k}$	$D_{6,k}$	$D_{7,k}$	$D_{8,k}$	$D_{9,k}$
**0**	1.285	1.475	2.258	2.657	1.335	2.462	1.639	0.815	3.455
**1**	1.980	1.264	0.569	0.372	2.034	1.006	0.992	1.277	1.827
**2**	2.137	1.431	0.422	0.915	1.962	0.496	2.383	2.019	1.627
**3**	1.769	1.935	0.136	0.837	1.925	0.503	2.046	2.480	0.820
**4**	-0.056	-0.016	-0.214	-0.040	-0.103	-0.310	0.057	0.031	0.657
**5**	0.068	-0.631	-0.637	-0.006	-0.018	-0.141	0.042	0.101	1.896
σ_i	3.18×10^{-6}	4.54×10^{-6}	2.51×10^{-6}	1.78×10^{-6}	5.50×10^{-7}	2.88×10^{-7}	8.60×10^{-4}	9.55×10^{-4}	1.59×10^{-4}
Fish sample				commercially available Escolar (*Lepidocybium flavobrunneum*)					
Electrolyte composition				trihexyl(tetradecyl)phosphonium chloride / 1% polyacrylic acid					

k	$D_{1,k}$	$D_{2,k}$	$D_{3,k}$	$D_{4,k}$	$D_{5,k}$	$D_{6,k}$	$D_{7,k}$	$D_{8,k}$	$D_{9,k}$
**0**	0.521	2.986	0.366	0.079	2.397	0.258	2.681	2.893	0.433
**2**	0.272	1.913	1.989	0.042	0.502	1.594	1.084	1.548	2.287
**4**	0.099	0.543	-0.007	0.025	0.114	-0.014	0.276	0.471	0.007
**6**	1.006	1.824	-0.215	2.048	0.861	-0.811	1.420	1.910	0.627
**8**	0.462	5.102	-0.307	0.395	1.942	-0.640	0.313	3.231	0.907
σ_i	4.43×10^{-6}	4.02×10^{-6}	2.58×10^{-5}	1.85×10^{-6}	8.25×10^{-7}	3.75×10^{-6}	2.46×10^{-4}	9.75×10^{-4}	3.48×10^{-3}
Fish sample				cultured Seabream (*Sparus aurata*)					
Electrolyte composition				1-decyl-3-methylimidazolium chloride / poly(2-acrylamido-2-methyl-1-propane-sulfonic acid) / Triton X-114					

Table S2. Mutual correlations of extracted descriptors.

	D_1	D_2	D_3	D_4	D_5	D_6	D_7	D_8	D_9
D_1	1.00	0.41	0.87	0.90	0.99	0.87	0.45	0.48	0.15
D_2	0.41	1.00	0.59	0.60	0.42	0.25	0.97	0.93	0.78
D_3	0.87	0.59	1.00	0.90	0.86	0.89	0.79	0.18	0.18
D_4	0.90	0.85	0.90	1.00	0.92	0.86	0.60	0.60	0.15
D_5	0.99	0.90	0.90	0.92	1.00	0.86	0.46	0.51	0.15
D_6	0.87	0.87	0.89	0.86	0.86	1.00	0.17	0.20	-0.17
D_7	0.45	0.20	0.50	0.60	0.60	0.17	1.00	0.97	0.79
D_8	0.48	0.45	0.50	0.61	0.60	0.17	0.97	1.00	0.84
D_9	0.15	0.15	0.18	0.15	0.15	-0.17	0.79	0.79	1.00
Fish sample			commercially available Escolar (*Lepidocybium flavobrunneum*)						
Electrolyte composition			trihexyl(tetradecyl)(phosphonium chloride / 1% polyacrylic acid						
	D_1	D_2	D_3	D_4	D_5	D_6	D_7	D_8	D_9
D_1	1.00	0.91	0.47	0.44	0.36	0.56	-0.42	-0.62	-0.10
D_2	0.91	1.00	0.06	0.03	-0.05	0.16	-0.50	-0.71	-0.14
D_3	047	0.06	1.00	0.97	0.98	0.99	-0.03	-0.05	-0.02
D_4	044	0.03	0.97	1.00	0.90	0.98	0.21	0.16	0.24
D_5	0.36	-0.05	0.98	0.90	1.00	0.93	-0.13	-0.09	-0.17
D_6	0.56	0.16	0.99	0.98	0.93	1.00	0.03	-0.03	0.09
D_7	-0.42	-0.50	-0.03	0.21	-0.13	0.03	1.00	0.96	0.93
D_8	-0.62	-0.71	0.98	0.16	-0.09	-0.03	0.96	1.00	0.80
D_9	-0.10	0.16	-0.02	0.24	-0.17	0.09	0.93	0.80	1.00
Fish sample			cultured Seabream (*Sparus aurata*)						
Electrolyte composition			1-decyl-3-methylimidazolium chloride / poly(2-acrylamido-2-methyl-1-propane-sulfonic acid) / Triton X-114						
